# Nonmetal Organic Frameworks Exhibit High Proton Conductivity

**DOI:** 10.1021/jacs.5c01336

**Published:** 2025-04-24

**Authors:** Megan O’Shaughnessy, Jungwoo Lim, Joseph Glover, Alex R. Neale, Graeme M. Day, Laurence J. Hardwick, Andrew I. Cooper

**Affiliations:** † Department of Chemistry, University of Liverpool, Liverpool L69 7ZD, United Kingdom; ‡ Computational System Chemistry, School of Chemistry and Chemical Engineering, University of Southampton, Southampton SO17 1BJ, United Kingdom

## Abstract

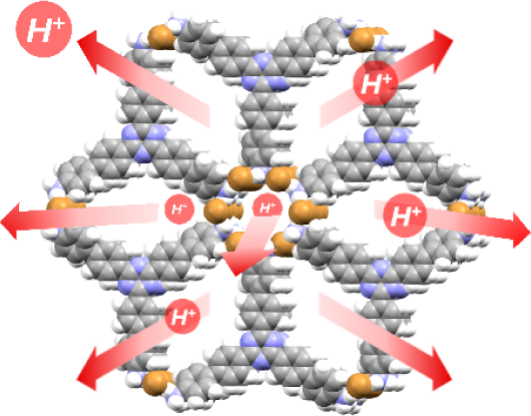

Porous materials, such as metal–organic frameworks (MOFs) and porous organic salts, are promising materials for proton conduction. Recently, we developed a new subclass of porous materials, isoreticular nonmetal organic frameworks (N-MOFs), which can be designed using crystal structure prediction (CSP). Here, two porous, isostructural, and water-stable halide N-MOFs were prepared and found to show good proton conductivity of up to 1.1 × 10^–1^ S cm^–1^ at 70 °C and 90% relative humidity. Changing the halides in these N-MOF materials affects the resulting proton conductivity, as observed in previous studies involving MOFs and lead halides. Although this is the first study of proton conductivity in N-MOFs, the bromide salt, **TTBT.Br**, shows a higher conductivity than most polycrystalline MOFs and porous organic salts, approaching that of Nafion.

## Introduction

Materials that exhibit high proton conductivity are important for the efficient conversion of chemical energy into electrical energy.^1,2^ Proton-conducting materials are used in fuel cells, electrolyzers, batteries, and sensors. Hence, the development of materials with high proton conductivity is needed to move us toward a hydrogen economy.

Metal–organic frameworks (MOFs) are a promising class of materials for proton conduction because of their high surface areas and tunable structures, which allow the incorporation of various functional groups or guests in the pores.^3^ Often, acid groups, such as phosphonates and sulfonates, are used to tune MOFs for proton conduction because they can act as proton transfer sites, thereby increasing performance.^4−6^ Alternatively, the pores of MOFs can be loaded with molecular acids, such as H_2_SO_4_ and H_3_PO_4_, to increase proton conductivity.^7^

Porous organic salts are a related class of molecular materials that have shown promise for proton conduction.^8−11^ Porous salts are produced by combining organic building blocks functionalized with acid and base groups.^12^ A range of acid–base combinations has been used to form salt frameworks; not all of these exhibit permanent porosity, but this is not a hard requirement for proton conduction. One of the best-performing porous organic salts for proton conductivity was reported by Bai et al., where a guanidinium arylphosphate showed a conductivity of 4.38 × 10^–2^ S·cm^–1^ (90 °C, 90% relative humidity, RH).^8^ Yan Teng and coworkers reported a series of porous salts in 2018: CPOS-1, which was permanently porous, showed one of the highest proton conductivities reported at that time (1.0 × 10^–2^ S cm^–1^, 60 °C, 98% RH).^12^ This team later incorporated H_2_SO_4_ into the pores of CPOS-1 to further enhance its performance to 1.4 × 10^–2^ S cm^–1^ (30 °C, 100% RH).^13^ Another method that has been used to enhance proton conductivity in porous organic salts is to form hybrid membranes with Nafion.^14,15^ Zhao et al.^16^ demonstrated the effectiveness of this method when they increased the performance of iHOF-8 from 5.02 × 10^–3^ S cm^–1^ (100 °C, 98% RH) to 1.6 × 10^–1^ S cm^–1^ (100 °C, 98% RH) by using Nafion with the material to form a membrane.

Recently, we reported a series of isostructural salts (nonmetal organic frameworks, N-MOFs).^17^ We showed that these materials exhibit properties that are, in many ways, like MOFs. For example, they can form isostructural families. These N-MOFs were formed using organic acids, such as hydrogen halides, whereby the halide ions are analogous to the metal nodes in MOFs. These porous molecular crystals were designed from first principles using crystal structure prediction (CSP),^18,19^ which showed that the porous phases observed experimentally were the thermodynamically most stable crystal packings available.

Based on these CSP calculations, we speculated that these porous N-MOFs might have good stability for practical applications, unlike many metastable frameworks that tend to collapse and form denser, nonporous structures.^17^ This was demonstrated initially for the application of iodine capture. This thermodynamic stability, coupled with the polar pore channels in these N-MOFs, prompted us to explore these materials as proton conductors.

## Results and Discussion

**Figure 1:**
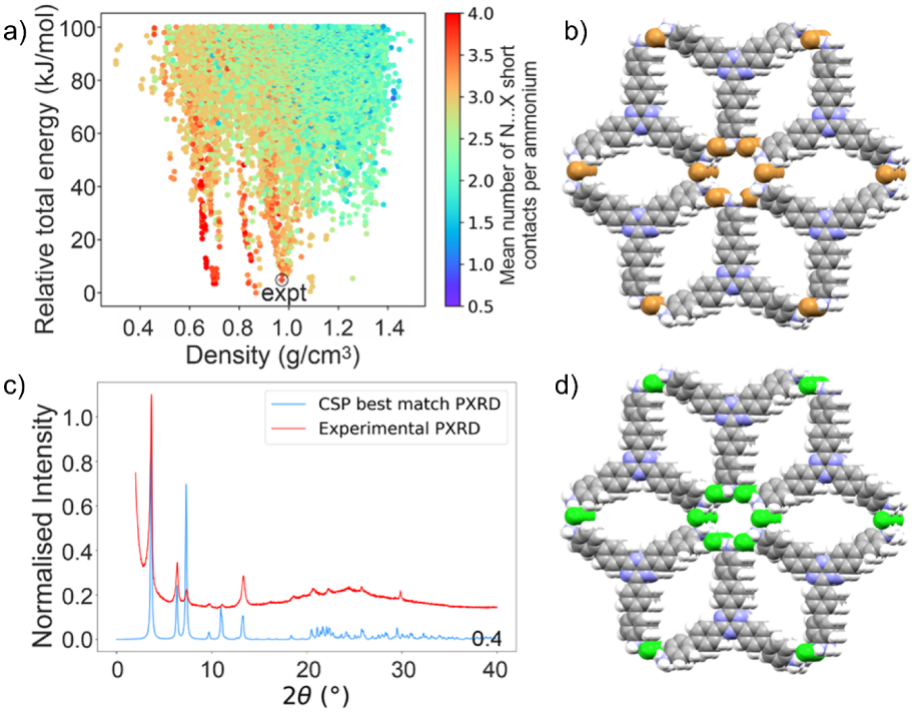
(a) Lattice energy landscape of predicted crystal structures for **TTBT.Br**, where each point corresponds to a distinct structure produced by CSP; data points are colored by NH_3_···halide close contacts. The predicted structure corresponding to the experimental structure is labeled in the image. (b) CSP space-filling packing model for **TTBT.Br** showing 1D polar salt pore channels. (c) PXRD patterns for the experimental material and low-energy CSP structure for **TTBT.Br**. (d) CSP space-filling packing model for TTBT.Cl showing 1D polar salt pore channels.

Of the three NMOFs reported in our previous study,^17^**TTBT.Cl TTBT.Cl** (4′,4‴,4⁗′-(1,3,5-triazine-2,4,6-triyl)tris[[1,1′-biphenyl]-4-amine] chloride) seemed most promising as a potential proton conductor because it is water stable and water insoluble, as well as being suggested by CSP to be the thermodynamically most stable structure. **TTBT.Cl** also adsorbs a substantial quantity of water (12.4 mmol g^–1^), and water sorption has been shown to improve high proton conductivity in some materials. Here, we also prepared the bromide analogue of **TTBT.Cl**, **TTBT.Br**. We chose to study this bromide analogue because previous reports for MOFs and lead halides have shown that conductivity can be tuned by varying the halides.^20,21^ CSP suggested that **TTBT.Br** would most likely crystallize like **TTBT.C**l (Figure 1a,b). The two predicted energy-structure landscapes have similar overall distributions of structures, including spikes corresponding to low-energy, porous structures that are stabilized by strong clustering of halide anions around the amine groups on TTBT. Many of the low-energy predicted crystal structures are common between the CSP landscapes of **TTBT.Cl** and **TTBT.Br** and one of the low-energy predicted structures for **TTBT.Br** being isostructural to the known crystal structure of **TTBT.Cl** (Figure 1c).

As for **TTBT.Cl**, attempts were made to grow single crystals of **TTBT.Br,** but the low solubility and rapid crystallization of this material made it hard to obtain suitable single crystals for single-crystal diffraction. The structure was therefore confirmed by comparing powder X-ray diffraction (PXRD) data with the predicted PXRD patterns obtained from CSP (Figure 1c). Based on the good agreement in peak positions in the PXRD and the agreement of CSP with experimental results in similar systems,^17^ we believe that the largest uncertainty in the predicted structure is in the precise torsion angles of the biphenyl TTBT arms.^17^

**Figure 2:**
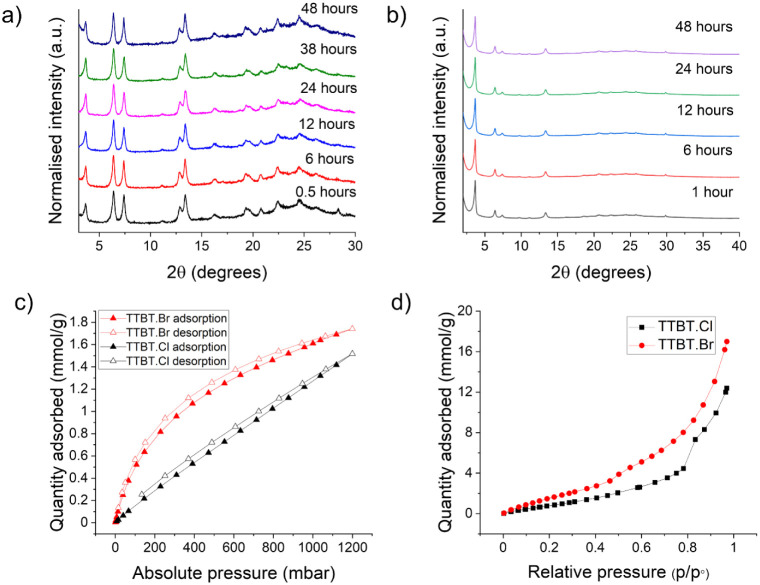
Stability of (a) **TTBT.Cl** and (b) **TTBT.Br** pellets over a 48 h period observed using PXRD. (c) CO_2_ isotherms at 273 K for **TTBT.Cl** and **TTBT.Br,** showing their permanent porosities. (d) Water isotherms for both N-MOFs at 298 K.

**Figure 3:**
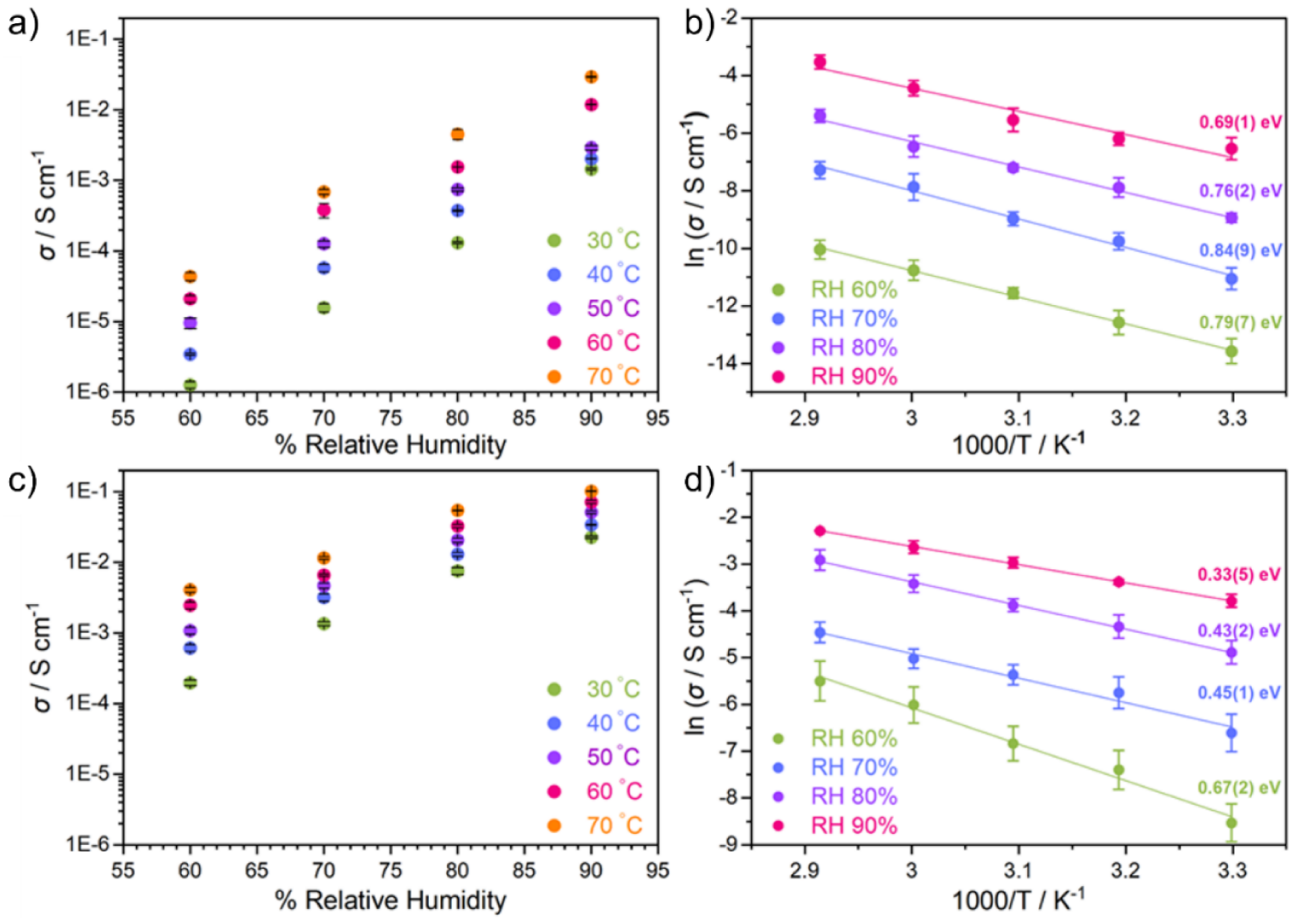
Proton conductivity of (a) **TTBT.Cl** and (b) **TTBT.Br** at different temperatures (30–70 °C) and humidity (60–90%), calculated based on bulk resistance from the Nyquist plot. Arrhenius plots for activation energies at different relative humidity for (c) **TTBT.Cl** and (d) **TTBT.Br**.

While the growth of single crystals proved challenging, the synthesis of TTBT.***X*** (*X* = Cl, Br) was easily scaled, and multigram quantities of material could be produced in 30 min by simple dropwise addition of the respective HX solutions into a solution of the TTBT linker in tetrahydrofuran. This resulted in the instant precipitation of **TTBT**.***X***, which was then collected by filtration. After drying the powders, they were pressed into 8 mm pellets using a pressure of 2 tons for 180 s, and their stability to water was tested using PXRD (Figure 2a,b). Both N-MOF samples showed good stability, so they were tested for their proton conduction performance. For the proton conductivity tests, **TTBT.Cl** and **TTBT.Br** were tested from 30–70 °C with relative humidity (RH) in the range of 60–90%. **TTBT.Cl** exhibits poor proton conductivity (10^–6^–10^–5^ S cm^–1^) under moderate-to-low humidity conditions (60% RH) (Figure 3a). However, under wetter environments, the proton conductivity (2.9 × 10^–2^ S cm^–1^ at 70 °C, 90% RH) is comparable to the more conductive organic salts reported so far (Table 1). The **TTBT.Br** material performs better, displaying high proton conductivities of 1.01(3) × 10^–1^ S cm^–1^ at 70 °C, 90% RH. These conductivities are one to 2 orders of magnitude higher than **TTBT.Cl** as measured under the same temperature/humidity conditions. Indeed, the proton conductivity of **TTBT.Br** is close to that of Nafion 117 (7.5 × 10^–2^, S/cm at 60 °C, 98% RH).^22^ As such, **TTBT.Br** shows the highest proton conductivity of any polycrystalline organic salt reported to date. Cao et al.^23^ recently reported a higher proton conductivity for a single crystal of a porous salt (iHOF-16; 0.388 S cm^–1^ at 80 °C, 98% RH), but this high value was measured along a single crystallographic axis rather than as a bulk measurement, as here.

**Table 1:** The Best-Performing Polycrystalline Organic Salts and MOF Proton Conductors Reported to Date^a^

Compound	Proton conductivity (S cm^–1^)	Conditions	Reference
Organic salts			
**TTBT.Br**	1.1 × 10^–1^	70 °C, 90% RH	This work
**TTBT.Cl**	2.9 × 10 ^–2^	70 °C, 90% RH	This work
UPC-H9	2.68 × 10^–2^	80 °C, 30% RH	(26)
CPOS-2	2.2 × 10^–2^	60 °C, 98% RH	(12)
HOF-GS-11	1.8 × 10^–2^	30 °C, 95% RH	(9)
TPMA-3F/MTBPS	1.34 × 10^–2^	90 °C, 95% RH	(27)
(C_5_H_3_SO_3_H)(CH_2_CH_2_NH)	1.18 × 10^–2^	60 °C, 97% RH	(28)
HOF-IPCE-1Pd-NH_3_	1.27 × 10^–3^	85 °C, 85% RH	(29)
Cage salt 1	1.1 × 10^–3^	30 °C, 98% RH	(10)
MOFs			
8HSA@MIL-101	3.06 × 10^–1^	85 °C, 98% RH	(24)
10HSA@MOF-808-(bSA)_2_	2.47 × 10^–1^	86 °C, 98% RH	(24)
[Ni(H_2_O)_6_][H_2_tcba]	2.1 × 10^–2^	80°C, 97% RH	(30)
Ti-dobdc–LiI	1.26 × 10^–2^	55 °C, 90% RH	(21)
[Zn(H2O)6][H_2_tcba]	1.1 × 10^–2^	80 °C, 97% RH	(30)
Ti-dobdc–LiCl	9.64 × 10^–3^	55 °C, 90% RH	(21)
(UiO-66-(SO_3_H)_2_)	8.2 × 10-^2^	80 °C, 90% RH	(32)
UiO-66-(SO_3_H)_4_	3.7 × 10^–1^	90 °C, 90% RH	(33)
CPO-27-NCSMA	1.0 × 10^–2^	60 °C, 70% RH	(34)
MIP-202(Zr)	1.1 × 10^–2^	90 °C, 95% RH	(35)
PFSA polymer membrane			
Nafion 117	7.5 × 10^–2^	60 °C, 98% RH	(13)

a A standard commercial grade of Nafion (Nafion-117) is included as a comparison.

The proton conductivity values for **TTBT.Br** are higher than those for equivalent polycrystalline pellets of iHOF-16 (2.11 × 10^–2^ S cm^–1^ at 100 °C and 98% RH). The conductivity of **TTBT.Br** is also higher than most MOFs, even those purposely tailored for their proton conductivity (Table 1). Increasing the degree of humidity significantly reduces the activation energy for proton transport in **TTBT.Cl** and **TTBT.Br** from 0.84 (9) to 0.69(1) eV and 0.67(2) to 0.33(5) eV, respectively (Figure 3b and d), highlighting that the transport mechanism is dependent on the water content in the pores,^24^ as for other MOFs and salts. Gong et al. showed previously that changing the halide from Cl to Br resulted in lower performance,^20^ while Levenson et al.^25^ showed the opposite effect. In both of those earlier studies, it was concluded that it was the halide with the highest water uptake that gave the better proton conductivity. Our results also show that proton is sensitive to the water content in the pores. For this reason, the dry-state porosity (Figure 2a) and water uptake (Figure 2b) in both N-MOFs were quantified.^31^

**TTBT.Br** absorbs more CO_2_ than **TTBT.Cl** in the dry state (Figure 2a). While it is hard to quantify, this might be because **TTBT.Br** has greater crystallinity. Indeed, we speculated earlier^17^ that **TTBT.Cl** was partially crystalline based on its CO_2_ uptake. Water isotherms were collected at 298 K for both materials (Figure 2b) and **TTBT.Br** showed a substantially higher water uptake of 17 mmol g^–1^, compared to 12.5 mmol g^–1^ for **TTBT.Cl**, in keeping with its higher CO_2_ uptake. This higher water content, coupled with greater crystallinity, could explain the higher proton conductivity of **TTBT.Br**, even without any anion effect. However, the strength of the ammonium halide salt interaction might also play a part in the conductivity performance, since Gong et al.^14^ proposed that chloride interactions were stronger than bromide interactions in MOFs, which boosted performance. In these N-MOFs, it is the bromide analogue, **TTBT.Br**, that has the stronger salt interaction with a Δp*K*_a_ of 13.1, while the **TTBT.Cl** interaction is weaker (Δp*K*_a_ of 10.7).

## Conclusion

In conclusion, we have shown that these first-generation N-MOF materials have proton conductivity that exceeds that of other organic salts and most MOFs reported so far (Table 1). The results show that the halide counterion in the N-MOFs can modify the proton conductivity, perhaps mostly because the **TTBT.Br** material is more crystalline and adsorbs more water. These N-MOF materials showed reasonable stability in water and no changes in the PXRD patterns observed over several days at room temperature, which is commensurate with the predicted thermodynamic stability of these organic crystals, as anticipated by CSP. At higher temperatures, the crystallinity began to decrease slightly after 2 days. These are the first examples of N-MOF materials for proton conductivity; as such, there is significant scope to further improve their long-term stability in water and improve proton conductivity performance.

## Supporting Information Available

Materials, synthesis methods, ^1^HNMR, IR, further proton conductivity details and computational details (PDF)

## List of Abbreviations

N-MOF: nonmetal organic frameworks
CPOS: crystalline porous organic salts
MOF: metal-organic framework
CSP: crystal structure prediction
