# Supplementary material for: Nonmetal Organic Frameworks Exhibit High Proton Conductivity
Source: J Am Chem Soc. 2025 Apr 24;147(18):15429–34. doi: 10.1021/jacs.5c01336 (PMC12063171; doi:10.1021/jacs.5c01336)
Supplement: Supplementary file 1 — ja5c01336_si_001.pdf [file ja5c01336_si_001.pdf]

Supplementary Information

Non-Metal Organic Frameworks Exhibit High Proton Conductivity

Megan O'Shaughnessy,[1] Jungwoo Lim,[1] Joseph Glover,[2] Alex R. Neale,[1] Graeme M. Day,[2] Laurence J. Hardwick*[1] and Andrew I. Cooper*[1]

[1] Department of Chemistry, University of Liverpool, Liverpool, L69 7ZD, United Kingdom

[2] Computational System Chemistry, School of Chemistry and Chemical Engineering, University of Southampton, Southampton, *SO17 1BJ*, United Kingdom

Materials and methods

Materials

All reagents were obtained from Sigma-Aldrich, Manchester Organics and Fluorochem and used as received. All gases for sorption analysis were supplied by BOC at a purity of ≥ 99.9%**NMR**

^{1}H spectra were recorded at 400 MHz on a Bruker Advance 400 NMR spectrometer. Chemical shifts are reported in ppm with reference to internal residual protonated species of the deuterated solvents used for ^{1}H and ^{13}C analysis.

Fourier Transform Infrared spectroscopy (FT-IR)

IR spectra were recorded on a Bruker Tensor 27 FT-IR instrument using an attenuated total reflectance (ATR) set-up for the neat solids.

Powder X-ray diffraction (PXRD)

Powder X-ray diffraction data were collected in transition mode on powder samples held on thin Mylar film in aluminium well plates on a Panalytical Empyrean diffractometer equipped with a high throughput screening XYZ stage, X-ray focusing mirror, and PIXcel detector, using Cu-Kα (λ = 1.541 Å) radiation.

Gas sorption analysis

Samples (>100 mg) of **TTBT.Cl** and **TTBT.Br** were activated for sorption measurements at 110 °C under reduced pressure for 16 hours. Carbon dioxide isotherms were collected up to a pressure of 1200 mbar on a micrometrics ASAP2020 volumetric adsorption analyser at 273 K. Water isotherms at 298 K were collected using Micromeritics 3flex volumetric adsorption analyser.

Proton conduction

For the proton conductivity measurement, **TTBT.Cl** or **TTBT.Br** powder (0.1 g) was pelletized via a hydraulic press (load and 1 minute; diameter: 8mm, thickness > 1mm). A T-shaped Swagelok cell was assembled with 2 platinum foils as blocking electrodes. The cell was connected to a potentiostat (Biologic, VMP3 series) with 2 probes (quasi 4 probes) and stored in a humidity chamber (Memmertt Celsius). Impedance spectra were obtained between 303 ~ 343 K and 60 ~ 90% of relative humidity within the 1Mhz ~ 1khz of frequency range (Sinusoidal perturbation: 100 mV). All spectra were measured three times for each sample with more than 4 hours of equilibrium time to stabilize the structure after water uptake. Each condition was measured with at least two different pellets.

Computational methods

Conformers of TTBT were obtained from a conformer search on the +4 cation using the Schrödinger MacroModel software package. The search uses the low-mode sampling method with energies calculated by the OPLS2005 force.[2,3] Each unique conformer was then re-optimized using density functional theory at the PBE0/6-311 G** level of theory with GD3BJ empirical dispersion correction as implemented in the Gaussian09 software package.[4,5] This yielded four unique conformers as shown in Figure S1. The small energy difference between all four conformers results in a similar chance of each forming a low-energy crystal structure, therefore all four conformers were used as starting points for our CSP calculations.

CSP was performed using the Global Lattice Energy Explorer program,[6] which uses low-discrepancy quasi-random sampling of crystal packing variables to produce uniform sampling of the lattice energy surface. Trial crystal structures were generated across 11 space groups, and their lattice energies were minimized until a target number of valid crystals (N_{valid}) was met (Table S1). Crystal structures were generated with one cation and four anions in the asymmetric unit cell. Rigid-molecule lattice energy optimizations were subsequently performed using the DMACRYS software.[7] The lattice energies were calculated using an anisotropic atom-atom energy model based on a revised version of the Williams 99 force-field, combined with atom-centred multipoles calculated from a distributed multipole analysis of the PBE0/6-311 G** density.[8,9] Multipoles up to hexadecapole on each atom were included, and the

polarizable continuum model was applied to the distributed multipole analysis to improve the electrostatic model using a dielectric constant of 3.0. Bromide parameters were taken from molecular dynamics studies of ionic liquids[10] and were deemed suitable based on tests performed in our previous work.[11] Duplicate crystal structures were removed from the final CSP landscape by calculating the similarities of simulated powder x-ray diffraction patterns.

Table 1. Space group sampling used in this work

Space group name	Space group number	N_{valid}
$P,1\ P\text{- }1, P2_12_12_1$	1, 2, 19	10,000
$Pna2_1, P2_1, C2, Cc, Pbca$	33, 4, 5, 9, 61	20,000
$P2_1/c, C2/c, R\text{- }3$	14, 15, 148	50,000

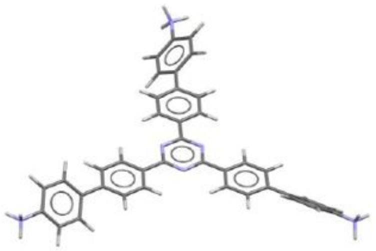

TTBT-1

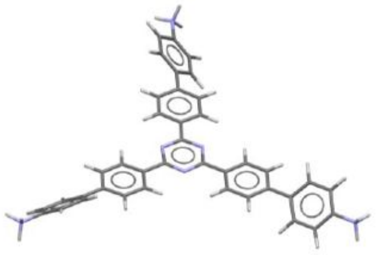

TTBT-2

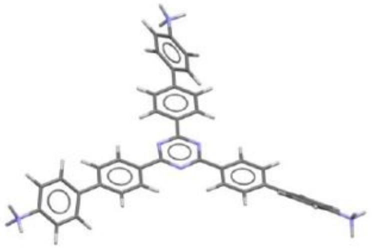

TTBT-3

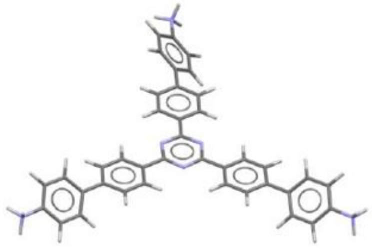

TTBT-4

Figure S1. The four TTBT conformers used in the CSP searches

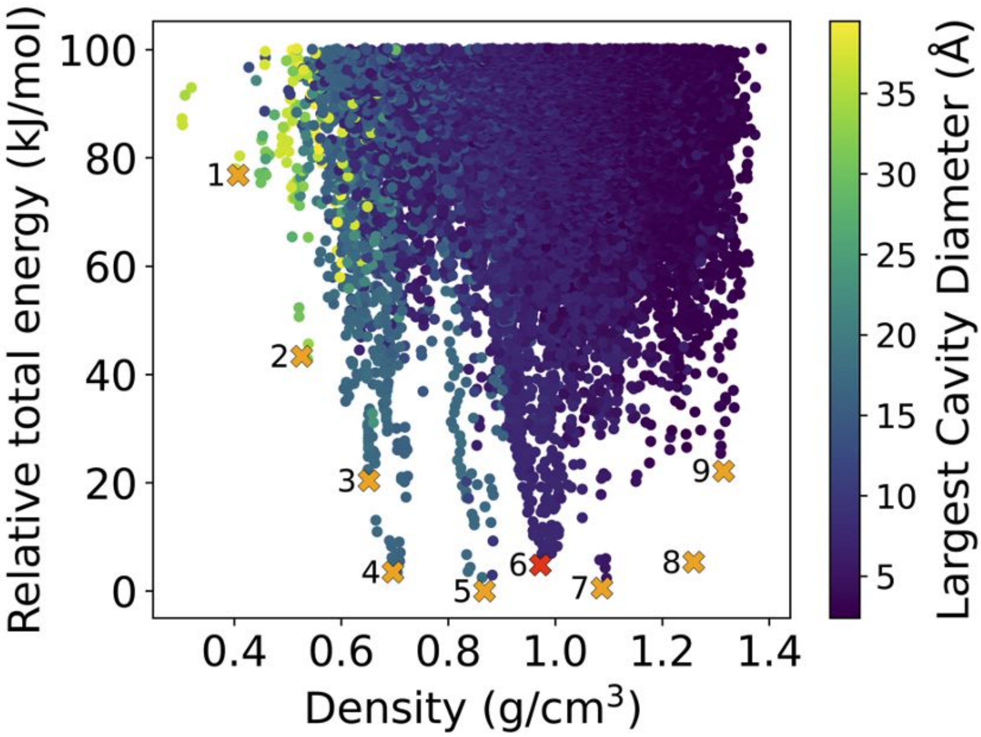

Figure S2. CSP energy landscape for **TTBT.Br**. A selection of low-energy structures (yellow crosses) are visualised below in Figure 3 corresponding to other polymorphs that may be experimentally accessible.

#	ID	Density	Relative	PLD	LCD

		(g/cm³)	energy (kJ/mol)	(Å)	(Å)
1	QR-148-87838-3	0.407	76.8	36.7	36.8
2	QR-15-14605-3	0.526	43.4	32.3	32.4
3	QR-4-11031-3	0.651	20.4	17.4	17.8
4	QR-33-39814-3	0.697	3.5	17.2	17.3
5	QR-148-46218-3	0.868	0.0	17.5	17.5
6	QR-15-221361-3	0.972	4.8	7.60	8.39
7	QR-61-39611-3	1.09	0.6	5.90	6.70
8	QR-15-169768-3	1.26	5.4	2.74	4.48
9	QR-15-182230-3	1.31	22.2	3.73	4.05

PLD – Pore limiting diameter; LCD – Largest cavity diameter

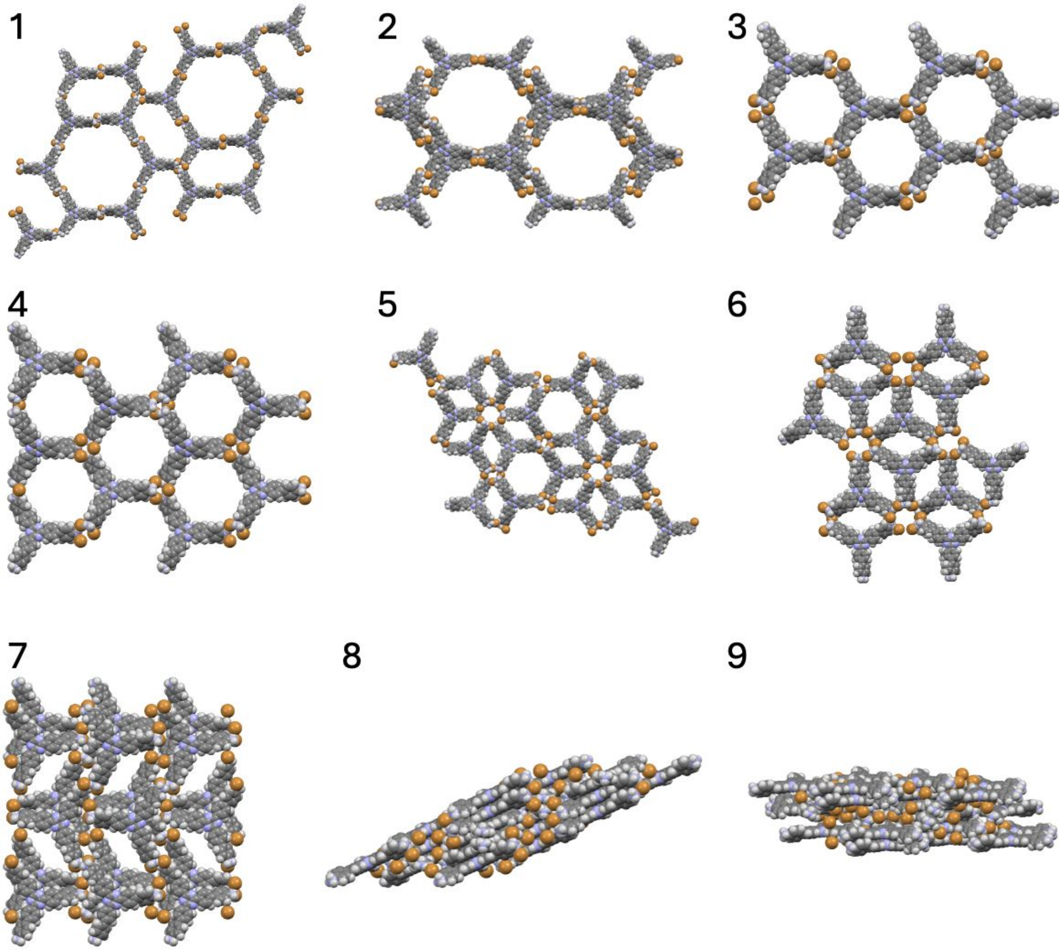

Figure S3. Examples of low-energy crystal structures that appear on the CSP energy landscape of TTBT.Br (see Figure S2, above).

Synthesis of TTBT.X

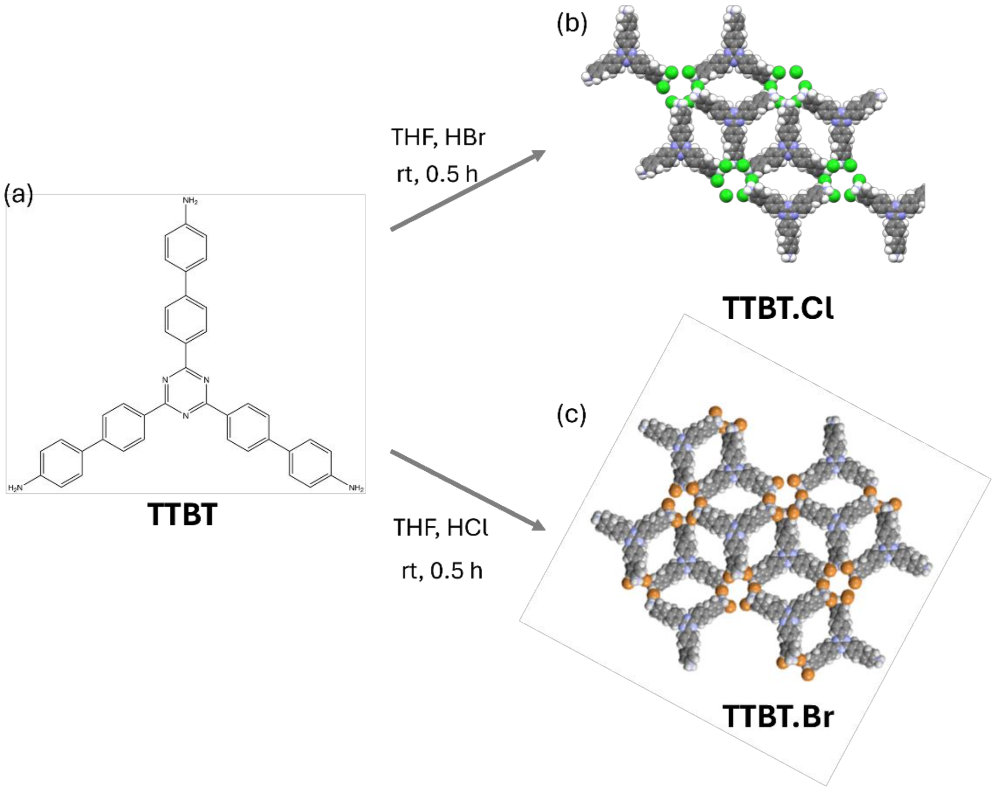

Scheme S1. (a) Chemical structure of 4,4',4''-(1,3,5-triazine-2,4,6-triyl) tris (1,1'-biphenyl) trianiline) (TTBT) ligand. The upper scheme shows the reaction conditions used to form **TTBT.Cl**, (b), represented as the sphere packing crystal structure. The bottom scheme shows the reaction conditions used to form **TTBT.Br**, (c), also represented as a sphere packing.

Bulk powders of **TTBT.Cl** were synthesised as reported previously.[1] **TTBT.Br** was prepared by dissolving TTBT in THF (5 mg/mL) and adding HBr (48% wt.% H_2O) with stirring until all amines were protonated. The mixture was left to stir for 30 minutes before being diluted with THF and filtered. The filtered solid was further washed with diethyl ether and THF. This brown solid was then dried under vacuum at 80 °C overnight (98% yield). ^{1}H NMR of **TTBT.Br** (Figure S5) (400 MHz, DMSO-d6), δ 8.78 (d, J = 8.5 Hz, 6H), 7.95 (d, J = 8.5 Hz, 6H), 7.80 (d, J = 8.5 Hz, 6H), 7.22 (d, J = 8.5 Hz, 6H).

NMR data

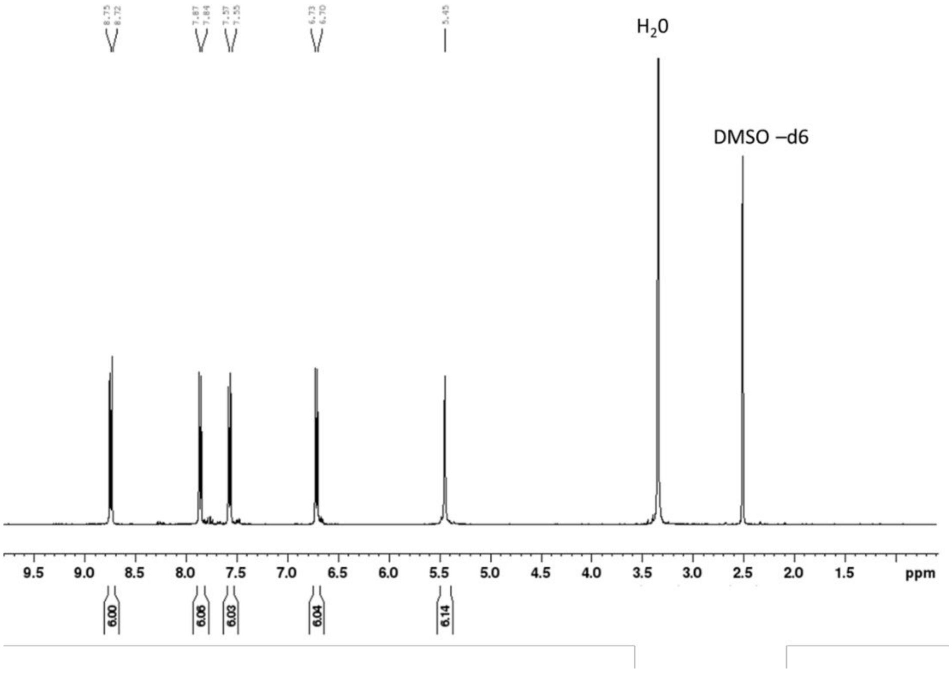

Figure S4. ^{1}H NMR of **TTBT.Cl** (400 MHz, DMSO-d6), δ 8.73 (d, J = 8.5 Hz, 6H), 7.85 (d, J = 8.5 Hz, 6H), 7.56 (d, J = 8.5 Hz, 6H), 6.71 (d, J = 8.5 Hz, 6H), 5.46 (s, 6H).

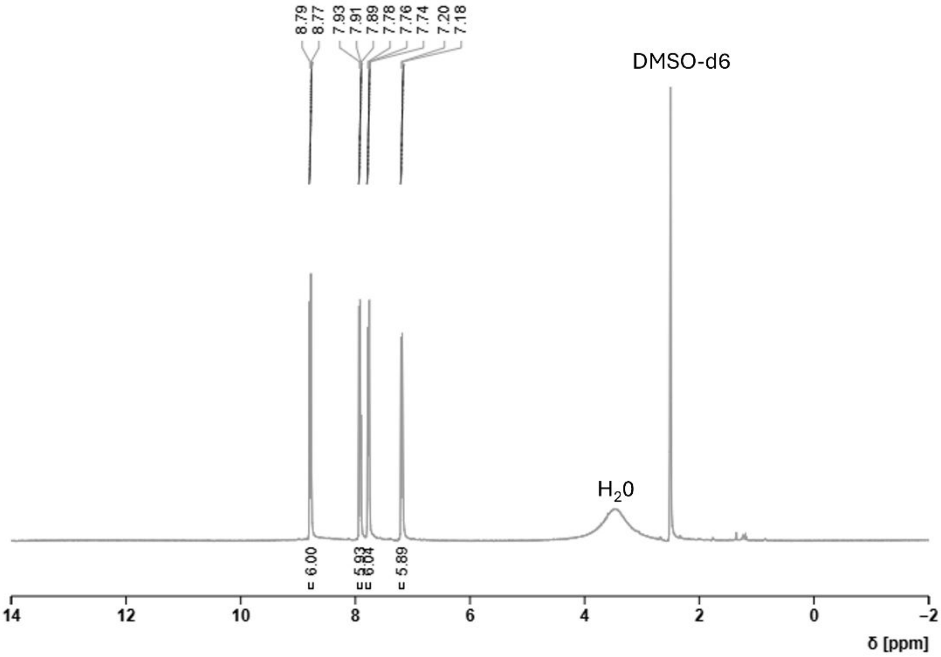

Figure S5. ^{1}H NMR of **TTBT.Br** (400 MHz, DMSO-d6), δ 8.78 (d, J = 8.5 Hz, 6H), 7.95 (d, J = 8.5 Hz, 6H), 7.80 (d, J = 8.5 Hz, 6H), 7.22 (d, J = 8.5 Hz, 6H).

PXRD data

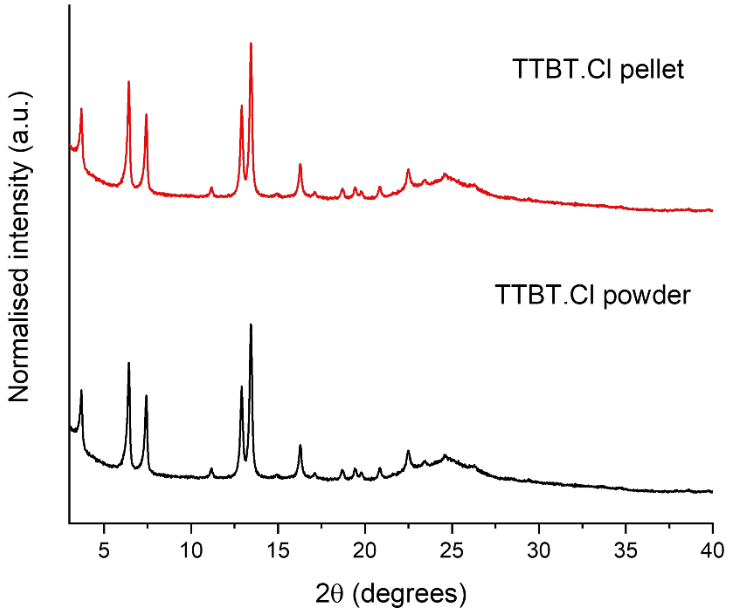

Figure S6. PXRD pattern for **TTBT.Cl** both as formed from the reaction (black line) and after it had been pressed into a pellet (red line) showing that the polymorph does not change upon pelletization.

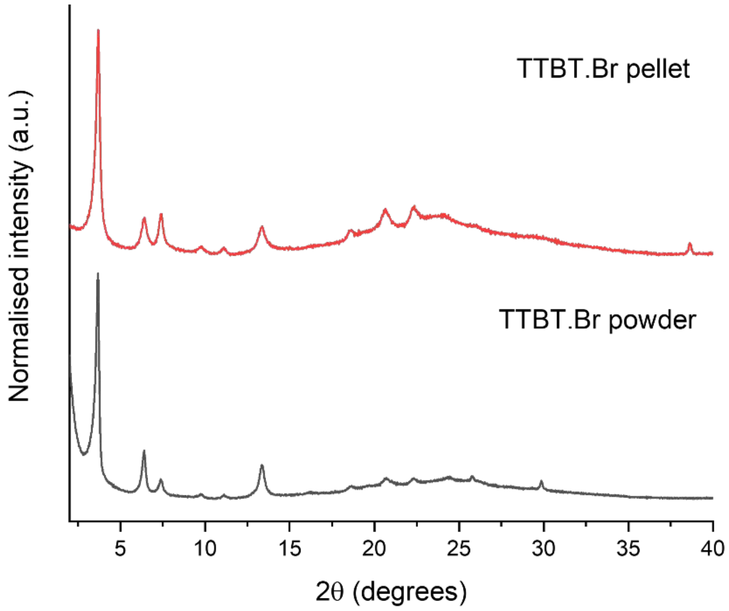

Figure S7. PXRD pattern for **TTBT.Cl** both as formed from the reaction (black line) and after it had been pressed into a pellet (red line), showing that the polymorph does not change upon pelletization).

Infrared spectra (IR)

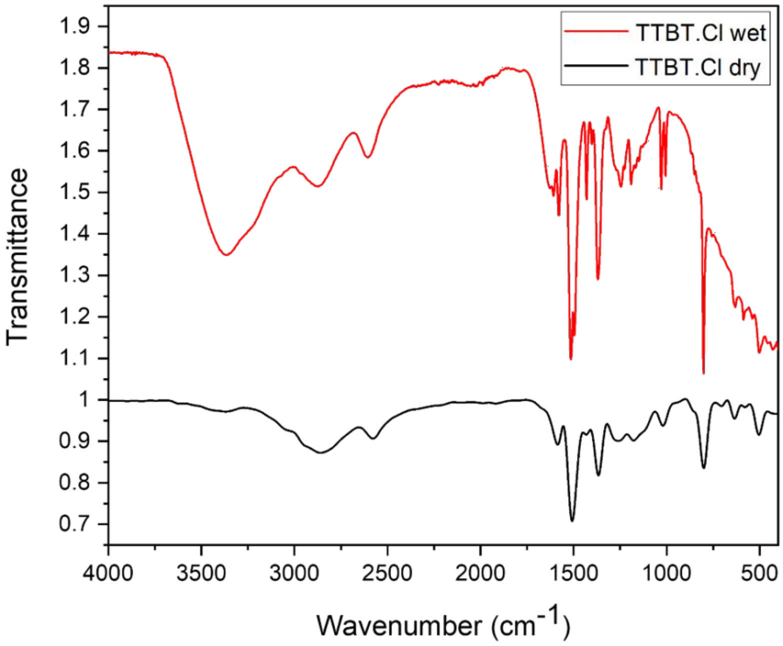

Figure S8. FTIR of **TTBT.Cl** both after activation (black line) and after being left submerged in water for 1 hour (red line).

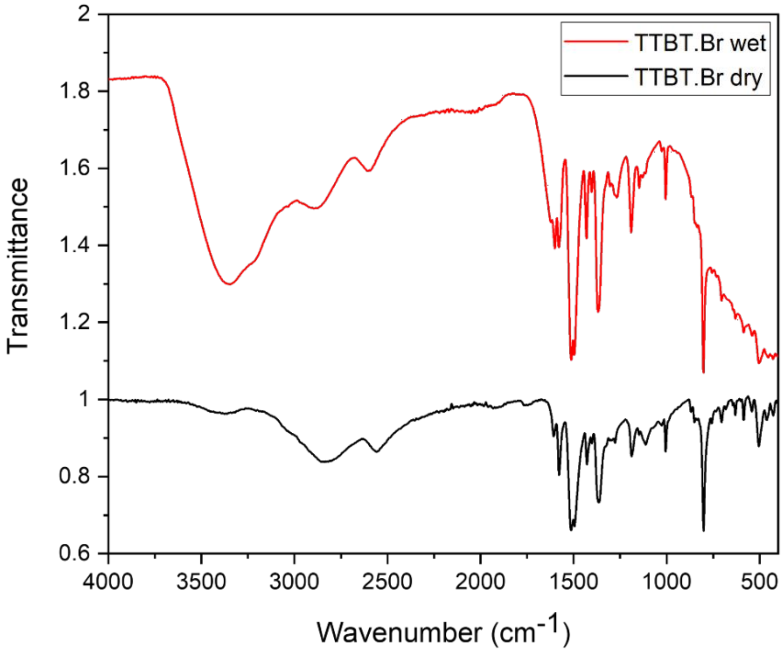

Figure S9. FTIR of **TTBT.Br** both after activation (black line) and after being left submerged in water for 1 hour (red line).

Proton conduction

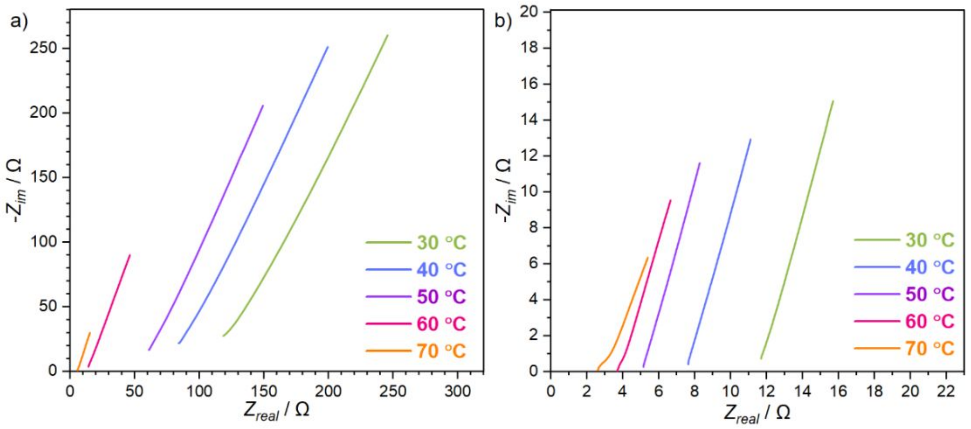

Figure S10. Nyquist plot for in the temperature range of 30 – 70 °C for (a) **TTBT.Cl** and (b) **TTBT.Br**.

SEM data

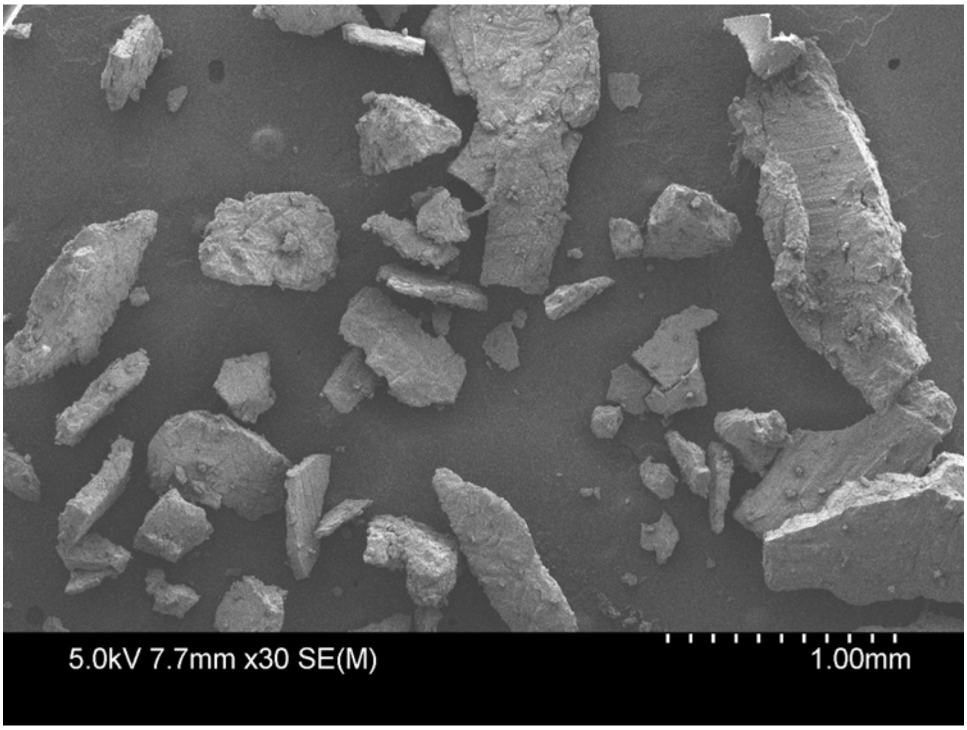

Figure S11. SEM image of **TTBT.Cl** as synthesized before being processed into pellets.

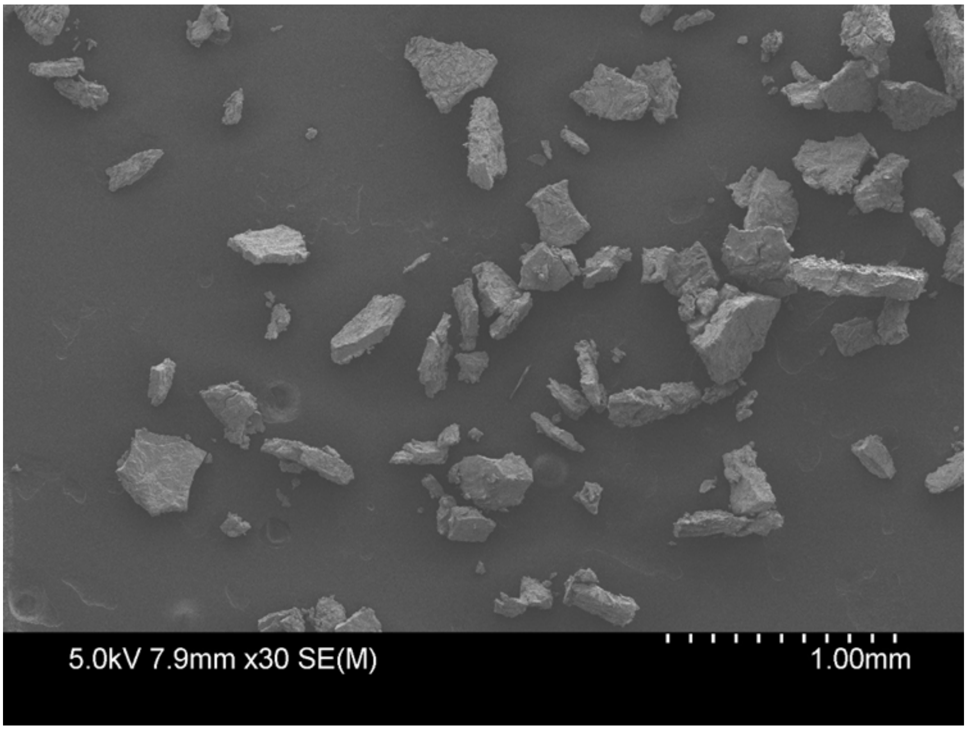

Figure S12. SEM image of TTBT.Br as synthesised before being processed into pellets.

Supporting References

1. M. O'Shaughnessy, J. Glover, R. Hafizi, M. Barhi, R. Clowes, S. Y. Chong, S. P. Argent, G. M. Day and A. I. Cooper. Porous isoreticular non-metal organic frameworks. *Nature*, **2024**, 630, 102-108.

2. Schrödinger MacroModel v.2017-4; Schrödinger LLC: New York, NY, 2017.

3. Jorgensen, W. L.; Tirado-Rives, J. The OPLS [optimized potentials for liquid simulations] potential functions for proteins, energy minimizations for crystals of cyclic peptides and crambin. *J. Am. Chem. Soc.* **1988**, *110*, 1657–1666.

4. Frisch, M. J.; et al. Gaussian v.09; Gaussian, Inc.: Wallingford, CT, 2009.

5. Grimme, S.; Ehrlich, S.; Goerigk. Effect of the damping function in dispersion corrected density functional theory. *J. Comput. Chem.* **2011**, *32*, 1456–1465.

6. Case, D. H.; Campbell, J. E.; Bygrave, P. J.; Day, G. M. Convergence properties of crystal structure prediction by quasi-random sampling. *J. Chem. Theory Comput.* **2016**, *12*, 910–924.

7. Price, S. L.; et al. Modelling organic crystal structures using distributed multipole and polarizability-based model intermolecular potentials. *Phys. Chem. Chem. Phys.* **2010**, *12*, 8478–8490.

8. Pyzer-Knapp, E. O.; Thompson, H. P. G.; Day, G. M. An optimized intermolecular force field for hydrogen-bonded organic molecular crystals using atomic multipole electrostatics. *Acta Crystallogr. B Struct. Sci. Cryst. Eng. Mater.* **2016**, *72*, 477–487.

9. Stone, A. J.; Alderton, M. Distributed multipole analysis. *Mol. Phys.* **1985**, *56*, 1047–1064.

10. Cruz, F. J. A. L.; Lopes, J. N. C.; Calado, J. C. G.; Minus da Piedade, M. E. A. A molecular dynamics study of the thermodynamic properties of calcium apatites. 1. Hexagonal phases. *J. Phys. Chem. B* **2005**, *109*, 24473–24479.

11. Cruz, F. J. A. L.; Lopes, J. N. C.; Calado, J. C. G.; Minus da Piedade, M. E. A. A molecular dynamics study of the thermodynamic properties of calcium apatites. 1. Hexagonal phases. *J. Phys. Chem. B* **2005**, *109*, 24473–24479.
